# Predicting Gene Expression Responses to Cold in *Arabidopsis thaliana* Using Natural Variation in DNA Sequence

**DOI:** 10.3390/genes16091108

**Published:** 2025-09-19

**Authors:** Margarita Takou, Emily S. Bellis, Jesse R. Lasky

**Affiliations:** 1 Department of Biology, Pennsylvania State University, University Park, PA 16802, USA; embellis@gmail.com (E.S.B.); jrl35@psu.edu (J.R.L.); 2 Department of Computer Science, Arkansas State University, Jonesboro, AR 72401, USA

**Keywords:** low-frequency variants, gene expression prediction, machine learning, regulatory elements, evolution, genotype–environment interaction

## Abstract

**Background/Objectives**: The evolution of gene expression responses is a critical component of population adaptation to variable environments. Predicting how DNA sequence influences expression is challenging because the genotype-to-phenotype map is not well resolved for *cis*-regulatory elements, transcription factor binding, regulatory interactions, and epigenetic features, not to mention how these factors respond to the environment. **Methods**: We tested if flexible machine learning models could learn some of the underlying *cis*-regulatory genotype-to-phenotype map to predict expression response to a specific environment. We tested this approach using cold-responsive transcriptome profiles in five *Arabidopsis thaliana* natural accessions. **Results**: We first tested for evidence that *cis* regulation plays a role in environmental response, finding 14 and 15 motifs that were significantly enriched within the up- and downstream regions of cold-responsive differentially regulated genes (DEGs). We next applied convolutional neural networks (CNNs), which learn de novo *cis*-regulatory motifs in DNA sequences to predict expression response to cold. We found that CNNs predicted differential expression with moderate accuracy, with evidence that predictions were hindered by the biological complexity of regulation and the large potential regulatory code. **Conclusions**: Overall, approaches for predicting DEGs between specific environments based only on proximate DNA sequences require further development. It may be necessary to incorporate additional biological information into models to generate accurate predictions that will be useful to population biologists.

## 1. Introduction

Adaptation is often a complex process that occurs via selection on traits and the underlying genetic mechanisms [1]. The genetic basis of ecologically important quantitative traits often involves mutations with effects on gene expression. In variable environments, the evolution of gene expression responses to environment (expression plasticity) is likely a critical component of adaptation [2]. These processes are often studied using evidence from variation in DNA sequence and mRNA abundance. For instance, the incorporation of transcriptomic information into genome-wide association studies (GWAS) can help identify genes controlling a specific phenotype [3]. Association or linkage mapping approaches can be used to map expression quantitative trait loci (eQTL), and allele-specific expression patterns can be used to infer the presence of *cis*- or *trans*-acting eQTL [4,5,6]. However, the integration of sequence and expression data in evolutionary quantitative genomics is hindered by gaps in our understanding of how DNA sequence influences expression. In particular, understanding the genetic basis of expression plasticity might be beneficial to applied biology given the importance of predicting organismal responses to environmental changes [7].

Gene expression responses to the environment differ among genes in the genome and among different natural genotypes due to DNA sequence variation. Differences in gene expression of a given gene among genotypes may be driven by sequence differences at other loci on different DNA molecules in the genome (*trans*-effects). At the same time, expression differences among genes and genotypes may arise from differences in sequences on the same DNA molecule (*cis*-effects). Researchers have documented cases where mutations in genes involved in environmental sensing [8,9] or in environmentally responsive genes that bind DNA sequences [10,11] have downstream effects on expression of other genes. Additionally, researchers have characterized how some *cis* mutations influence DNA binding by environmentally responsive transcription factors, ultimately altering environmentally responsive expression [12,13,14]. Transcription factors that tend to show expression responses to environment have been documented, and the DNA sequence motifs they bind to have been roughly characterized [15]. However, across the genome there are many interacting transcription factors that often respond to a single environmental stressor, but this knowledge is currently too limited to predict how genetic variation in DNA sequence influences genome-wide expression responses to environment.

The distribution of a large number of *cis*-regulatory motifs across the genome is associated with evolutionary and genomic properties, thus allowing the investigation of those properties [16,17,18]. Genome-wide expression profiling in association mapping or linkage mapping populations, as well as allele-specific expression studies, have revealed that *cis* expression quantitative trait loci (*cis*-eQTL) are abundantly segregating within species. *Cis*-regulatory mutations have been of particular importance for evolutionary biology because some have suggested they may be less likely to be deleterious than mutations in transcription factors, which are expected to be more pleiotropic [19,20]. Relatedly, the possibility of easier dissection of the genotype-to-phenotype map for *cis*-eQTL (versus *trans*-acting mutations likely influencing multiple genes’ expression) combined with the abundance of *cis*-eQTL may be an opportunity to build a more integrative understanding of quantitative trait genetics.

Machine learning methods in fields such as genomics and population genetics can help navigate increasingly bigger datasets and reveal complex patterns [21]. Especially in gene regulation and evolution, deep learning approaches can have an advantage over traditional methods in decoding enhancer grammar of gene regulation, as these models can learn complex *cis*-regulatory rules in a precise manner not biased by current knowledge [22] and can outperform more traditional methods, like clustering by k-mer or linear regression, in predicting gene expression [23]. For example, training a machine learning model on the imputed *cis* haplotypic information of RNA expression, Giri et al. (2021) [24] managed to train a model without incorporating the impact of *trans*-effects on gene expression variation in maize. This prediction was less variable than those using all SNPs and had an increased accuracy for predicting within-population variance [24]. Random forest models successfully classified protein-coding vs. non-protein-coding genes in maize [25]. Moore et al. (2021) [26] showed how known regulatory sites of *A. thaliana* and transcription factor (TF) interactions can be used in machine learning to predict clusters of gene co-expression related to stress response [27]. Similarly, in *A. thaliana*, information on TF binding sites has been harnessed to predict differential gene expression under stress, such as response to Fe [26], high salinity [28], and combined heat and drought stress [29]. However, in all of these cases, the authors used additional omics information during deep learning, such as chromatin accessibility or co-expression clusters, data that may be unavailable in many systems. There is increasing evidence that genetic diversity in the *cis* elements of plants can be maintained by selection [30]. Deep learning algorithms could be used for genome-wide identification of deleterious and adaptive mutations, which is a prerequisite for the genetic improvement of crops [31]. Using upstream and downstream genetic coding information could improve the prediction of the role of *cis*-regulatory elements in differential gene expression, in contrast to using only upstream regions in maize [32], especially when more than one genotype was included in the analysis [33]. However, those methods do not capture distal regulatory elements well, if at all [34], and the source of variability can impact their efficiency [35].

In *A. thaliana*, whose importance in evolution and ecology research has been growing over the years [36], the conserved noncoding sequences are mostly concentrated close to the coding region and impact gene regulation [37], as *cis*-regulatory variants are usually in linkage with the expressed transcript [16,38,39]. Even though there are conserved gene expression responses between species and populations, the increased genetic diversity within populations or species can cause phenotypic variance. Genes whose expression is affected by genotype–environment interactions (GxE) tend to show allele frequency correlations with climate and have elevated genetic variation in stress-responsive transcription factor binding sites [40]. Known TF binding site motifs of *A. thaliana* have been used to identify putative binding sites and then train a deep model for predicting *cis* changes in tomato [41].

Our aim was to use genetic variation among genes within individuals and among genotypes in the regions upstream and downstream of genes to predict expression response to specific environments in *A. thaliana* and assess the method’s potential contribution to understanding evolution of GxE. We aimed to predict in both a more general sense than other studies by using the sequences instead of known TFBS and in a more restrictive sense by classifying gene expression in specific environments within one species. For the analysis we used a published dataset on differentially expressed genes (DEGs) during cold acclimation of diverse *A. thaliana* genotypes [42]. Response to cold stress not only has some well-characterized signaling pathways, transcription factors, and transcription factor binding sites but has also been connected to adaptation to local environments [43,44,45]. Hannah et al. (2006) [42] had identified approximately 1500 up-regulated and down-regulated DEGs out of around 20,000 genes for multiple accessions as a response to cold acclimation and stress (Table S1). Here, we focused on the five *A. thaliana* accessions *Can*, *Col-0*, *Cvi*, *Ler*, and *Rsch*, for which the pseudogenomes from whole-genome resequencing data are published [46]. We included multiple genotypes in the analysis in order to capture some of the natural genetic diversity and potential GxE responses.

We first tested whether there are specific sequence motifs enriched along those regions of the differentially expressed genes that would suggest *cis* regulation drives some aspect of cold expression response. We determined if these motifs were related to known stress-responsive transcription factors (TFs). Then, we tested whether several naïve deep learning models could predict expression response, without a priori designation of important motifs, by using only the sequences upstream and downstream of genes.

## 2. Materials and Methods

### 2.1. Transcriptome and Genome Data

We used the publicly available dataset of differentially gene expression during cold acclimation by [42]. In brief, the authors used multiple accessions of *A. thaliana* to test their cold acclimation potential. They found that approximately 4000 genes respond to cold acclimation, indicating regulatory pathways being involved in their control. Out of the accessions used in the original study, we used five accessions, i.e., *Col-0*, *Ler*, *Cvi*, *Can*, and *Rsch* (Table S1), whose pseudogenomes are available at https://1001genomes.org/ (accessed on 1 November 2021). For comparison with a potentially simpler transcriptomic change, we used differential expression of 1280 genes in the *Col-0* background of a *tt8* knockout line. The *tt8* gene regulates biosynthesis of anthocyanins, which we reasoned would be a narrower regulatory network than that involved in environmental responses [47].

### 2.2. Searching for Sequence Motifs Using STREME

Transcription factors bind DNA in a sequence-specific manner, but not all sequences are known, nor is the exact relationship between sequence and binding affinity known. For this reason, we began with a de novo search for sequence motifs in the upstream and downstream regions of the coding sequence, as those often contain such signals. We used samtools faidx v1.10 [48] to extract the 1k bp upstream and downstream regions of each gene present in the dataset, including the first and last parts of the coding region, as TFBS often occur there. Specifically, we extracted 500 bp upstream and 500 bp downstream of the gene’s TSS for the upstream regulatory coding region and 500 bp upstream and 500 bp downstream of the gene’s end transcriptional site for the downstream regulatory coding region. We then used the software STREME v5.5.8 [49] to calculate significantly enriched ungapped motifs within the set of up- and down-regulated DEGs in relation to their presence in non-DEGs. For both groups, we used a randomly down-sampled set of the non-DEGs as a control, and we stopped the search for motifs when we failed to identify motifs with high enough confidence (e-value > 0.05) three times in a row. This way, the statistical significance of each motif was determined based on whether it can classify sequences as DEGs or non-DEGs.

Because sequence motifs may have a variety of causes and functions, and not all are likely TFBS, we next sought to determine whether motifs discovered de novo by STREME specifically correspond to known transcription factor binding sites. We searched for significant overlaps of at least 5 bp between the discovered enriched motifs and the JASPAR 2022 CORE [50,51] plant transcription factor binding sites database with MEME v5.5.8 suite’s online tool TOMTOM v5.5.8 [52]. Significant overlap was determined by scoring the match of all possible alignments between sequence and known motif and summing those scores to produce *p*-values, q-values, and e-values.

### 2.3. Random Forest Analysis

To determine whether known transcription factor binding sites regulate the response to cold, we attempted to predict up-regulated or down-regulated DEGs vs. non-DEGs based on their presence or absence in the regulatory regions. We used a dataset [53] of TF putative binding sites 1000 bp upstream of each gene, which were predicted from *A. thaliana* using the JASPAR2018 Bioconductor package (JASPAR) and then matched to the aligned regions of *A. thaliana*. We treated the count of each TF binding site per gene as a separate feature and additionally determined the total number of TF binding sites per gene. Taken together, these descriptors of genomic variation provided a total of 413 features, of which we could assess the predictive impact on gene regulation.

We trained a random forest model for *Col-0* using the ranger v 0.16 library in R [54] with up-regulated DEGs in response to cold versus non-DEGs as the dependent variable and all compiled features of gene regulation as predictor variables. We set the number of trees of the forest to 500, the number of variables to possibly split at each node (mtry) to 200, and the impurity mode to impurity_corrected. From the trained random forest, we extracted the relative importance of each predictor variable and the out-of-bag prediction error, which is informative about the predictive power of the model. Finally, we estimated a *p*-value for each predictor variable using the “Altmann” method with 100 permutations. We repeated the random forest analysis for all down-regulated DEGs. We estimated the prAUC in the R pROC v1.18.5 library using the function prauc and determined its significance by permuting the gene identity 100 times for each set of variables.

### 2.4. Training Convolutional Neural Networks

We employed a more naive machine learning approach to determine whether we can predict DEGs using regulatory regions. We trained convolutional neural networks (CNNs) to identify a model that would predict whether each gene was differentially expressed in the stress conditions relative to the control. We prepared the dataset by separating the genes into a training and test set for each accession, incorporating information about the gene families to which they belonged [55]. Because sequences may carry spurious associations with expression that are due to shared evolutionary history, we followed the method of [55] and split genes into gene families and then tested predictions in gene families not used for training.

In the end, we used 68,979 (80%) genes, from which 20% (13,796 genes) was always retained as the validation set and 17,475 (20%) as the test set out of the total 86,454 genes across all five genotypes. Each unique gene could be present multiple times in the dataset, representing each accession. The proportion of up- and down-regulated DEGs to non-DEGs was approximately 1:11 in both sets (Table S1). For each gene, we used the samtools faidx v1.10 [48] to extract the 1.5k bp upstream and downstream regions of each gene from the published pseudogenomes, using custom scripts. Specifically, we extracted the 1000 bp upstream and 500 bp downstream of the gene’s transcriptional start site (TSS) for the upstream regulatory coding region and 500 bp upstream and 1000 bp downstream of the gene’s transcriptional termination site for the downstream regulatory coding region. Those sequences were converted into one-hot encoding of each nucleotide in Python v3.6, to be used as input for the training and testing of the CNN models. We then created labels needed for binary classification of DEGs during training by scoring up-regulated DEGs as 0 and non-DEGs as 1. As the number of up-regulated DEGs was much smaller than non-DEGs, we oversampled the up-regulated DEGs with replacement to a ratio of 1:1 between the two groups. This process was repeated for training models to classify genes as down-regulated DEGs and non-DEGs.

We used the prepared inputs and labels of the training set to perform a grid search of tuning parameters, as we wanted to identify both the models with the best prediction of our dataset and how different tuning parameters influence model accuracy. The grid search was performed on a total of 1344 combinations of hyperparameters [55] by training each model for 50 epochs using the Python library tensorflow-gpu v2 [56]. The tuning parameters included the following: the number of convolutional layers; the filter of each convolutional layer; the width of each convolutional layer; the pool width and stride; the number of dense layers and their units; and the dropout rate. A list of specific values used to create all different combinations is provided in Table S2. During each training phase, we used prAUC values of the validation set to tune, select, and save the best-trained model for further evaluation. For each trained model, we extracted loss, accuracy, and probability-based accuracy values under the curve (prAUC) of training, validation, and testing of each model to compare the accuracy of models. Moreover, we estimated the proportion of correct predictions of each class within the test set so that we could assess the potential overfitting of each model to predict within one class or the other. We estimated this within-class accuracy as the number of times that the class was predicted correctly divided by the total number of genes belonging to this class. In this context, “class” refers to up-regulated or non-DEG genes. The absolute difference in within-class accuracies, referred to in the text as prediction accuracy between the two classes, was also estimated. We repeated this process for all down-regulated DEGs.

### 2.5. Determining Factors Influencing Accuracy of CNNs

To determine the degree to which predictions relied on upstream sequence versus both up- and downstream sequences, we repeated the above-described analysis for the regulatory regions of all accessions by using only the upstream regions.

Because genetic variation among genotypes in expression can be driven by *trans*-effects (from other loci; far from the gene), we sought to control for some of this variation. In particular, we focused on eliminating variation at the large-effect CBF transcription factors that are involved in adaptation to different temperatures. The CBFs, or *C-REPEAT BINDING FACTORs*, are a set of 3 transcription factors that regulate cold acclimation and segregate for loss of function variation at high frequency in *A. thaliana*, with loss of function variants associated with warmer climates [45,57]. Hannah et al. (2006) [42] previously showed with our dataset that intact CBF function explained a substantial portion of variation in cold response transcriptomes, and Des Marais et al. (2017) showed that genetically variable cold responses were enriched in central locations of co-expression networks. First we trained and evaluated CNN models on those lines (*Can* and *Cvi*), which have all 3 CBF copies (*CBF1*, *CBF2*, and *CBF3*) differentially expressed between the experimental conditions [45,58] to test the training when all copies are expressed significantly. Moreover, we trained in those lines (*Rsch*, *Col-0*, and *Ler*), which have no intronic or other SNP differences at these loci, in order to eliminate genetic variation contributing to the model training.

To determine the role that genetic variation among genotypes versus only among genes within a genotype plays, we trained models with only *Col-0* genes as the set. We repeated the training while also oversampling the dataset to be the same size as for all five accessions combined to control for underpowered training due to the smaller dataset.

To assess whether models may have been hindered by factors such as model architecture or data complexity, we introduced a “spike” of 5 bp in the upstream region of up-regulated DEGs. This way we created a positive control that can help validate the capability of the models to recognize the up-regulated DEG group. We replaced the first 5 bp of the up-regulated DEGs with the sequence AAGGG. Then, we evaluated each trained model in two dimensions, both based on their accuracy of predicting the evaluated set and on their accuracy when predicting non-spiked data.

### 2.6. Evaluating and Interpreting of Model Training

We compared the area under the precision-recall curve (prAUC), cluster accuracy, and the difference of the two classes’ accuracy with a Kolmogorov–Smirnov test using the function ‘ks.test’ in R v4.1.2. To see which hyperparameters had a significant influence on those CNN performance metrics of the spiked and non-spiked datasets, we ran a generalized linear mixed model with all the hyperparameters as fixed effects and the models’ loss as a random effect. We dropped from the model all the fixed effects that had no significant impact on the prAUC. Therefore, the final model for the absolute difference between class prediction accuracy is expressed as follows [59,60]:

diff ~ cl * dl + pl + du + (1|fl)

where diff refers to the absolute difference between class prediction accuracy; cl stands for the number of convolutional layers; dl is the number of dense layers; pl is the pool stride; du represents dense units as fixed effects; and fl is the first convolutional layer’s filter. The residuals followed a Gamma distribution. The final model for the prAUC of the test set is as follows:

diff ~ cl * dl * pl + dr + (1|fl)

where dr is the dropout rate, and the residuals’ distribution was binomial. We tested the same models in the original complete dataset. Generalized linear mixed models were performed using the package lme4′s v1.1-35 function glmer [59], and *p*-values were extracted using the function Anova from the R package car v3.1 [60].

For each type of CNN training, we selected a model with the best performance during the testing phase in order to understand the architectures that could offer the best prediction of both classes. We selected models that had a prAUC value on the test set of at least 0.7 and those that did not overfit into predicting one class of DEGs. To do so, we estimated the absolute difference of the accuracy of predicting each class in the dataset. We used the same criteria for all cases.

Once we had selected the model parameters that comprise the architecture that best predicted the DEG class, we evaluated the significance of the result and excluded the possibility that it was the result of learning from spurious sequences by permuting the label identity of each gene 100 times and re-training using the same model architecture. We estimated the *p*-value by counting the cases that had better performance based on both the prAUC and the class accuracy difference and dividing by 100.

All results were visualized in R v4.1.2 [61].

## 3. Results

### 3.1. The Presence of Enriched Motifs in the Up-Regulated DE Gene Sequences Suggests Their Potential Contribution to Environmental Responses

**Figure 1:**
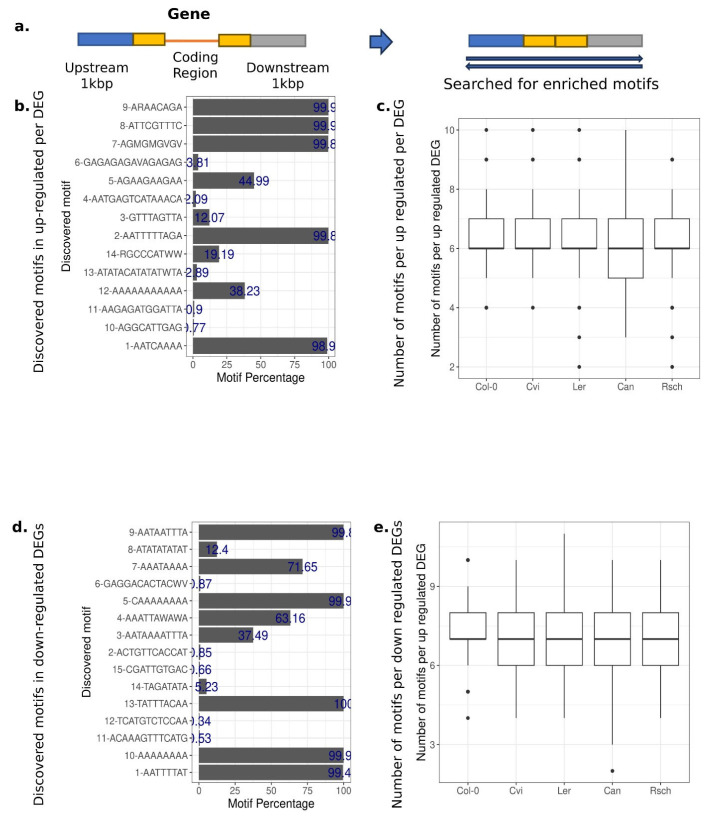
Significantly enriched motifs within the regulatory regions of the up- and down-regulated DEGs. (**a**) For each up- or down-regulated gene across all five *A. thaliana* accessions, we extracted 500 bp upstream (blue block) and downstream regions (gray block). We also extracted the first 500 bp after the transcriptional starting site and 500 bp before the transcriptional termination site (yellow blocks) for a total of 1k bp per gene (in order to br included in the motif search). (**b**,**d**) The discovered motifs in the upstream and downstream regions of up- or down-regulated DEGs and how frequent they are in the DEG sequences with STREME. (**c**,**e**) The distribution of discovered motifs in the upstream region of up-DEGs or down-DEGs per accession.

We searched for motifs enriched along the putative regulatory regions of the up-regulated DEGs in comparison to the non-DEGs to assess the possibility of patterns in the regulatory regions associating with the differential expression status of each gene. We used STREME [49] to search for de novo enriched motifs within the upstream and downstream regions of all genes of interest (Figure 1a). When we searched for enriched motifs only within the sequences of up-regulated DEGs for the reference genotype, *Col-0*, we discovered nine motifs. In contrast, we identified fourteen motifs significantly enriched within the up-regulated DEGs of all five accessions, suggesting greater power with more genotypes (*p* < 0.05). The median discovered motif length was 10 bp (Table S3), and the majority of the discovered motifs were significantly enriched in high frequency in our dataset. Specifically, five of the fourteen discovered motifs were present in more than 98% of tested DEGs’ regulatory regions, even though a few rare ones with less than 1% frequency were also identified (Figure 1b). When we looked at which genotypes each discovered motif was significantly enriched for at least once, the average number of motifs per up-regulated DEG was similar between the genotypes (approximately six motifs per gene per genotype; Figure 1c).

There was a similar number of enriched motifs among the down-regulated DEGs. In total, 15 motifs were discovered that were significantly enriched in all genotypes (*p* < 0.05; Table S4). Five of those motifs were present in more than 99% of tested DEG sequences for enrichment (Figure 1d), with an average of six discovered motifs per tested DEG sequence (Figure 1e).

When we cross-referenced all the motif sequences we identified as enriched in up- and down-regulated DEGs in a database of experimentally defined TFBSs [50], the discovered motifs had a significant overlap with 135 and 143 known TFBS, respectively (*p* < 0.05). The most common TFBS across the discovered motifs were members of the DOF family, with CDF5, DOF1.7, and DOF5.1 binding sites being present in three and four of the total discovered motifs in the up-regulated and down-regulated DEGs, respectively. The most common discovered sequences overlapping with the members of the DOF family were ARAACAGA in up-regulated and CAAAAAAAA in down-regulated DEGs. Members of the DOF family of transcription factors have been characterized as being involved in cold response [62]. Moreover, *PISTILLATA* (PI), a member of the MADS-box factors family MIKC, and the C2H2 zinc finger factor SGR5 binding sites were present in three and four of the discovered motifs in the up-regulated DEGs, respectively. There was an overlap of the most common TFBS across the discovered motifs of the up-regulated DEGs and the down-regulated DEGs (Table 1). Therefore, within the promoter regions of the up- and down-regulated DEGs, there are motifs present that could be potentially informative for classifying the gene expression.

### 3.2. Known Transcription Factor Binding Sites Within Col-0 Do Not Accurately Predict the Expression Response to Cold Using Random Forests

Given the many known and documented transcription factor binding site motifs that exist for different transcription factors (many of which are known to be involved in cold response) and given that we found TFBS motifs in the cold DEGs, we asked if these TFBS could predict genome-wide expression response to cold. We used a dataset of known TFBS in putative promoters 1k bp upstream of genes in the *A. thaliana* accession *Col-0* [53] to test whether they can predict DEGs using random forests classification trees (Figure S1). For this analysis, we also split the genes based on up-regulation and down-regulation and compared each group to all non-DEGs. Even though both random forest models correctly predicted the gene regulation status for approximately 80% of the genes, the prAUC of each of those models was low. The probability AUC-ROC (prAUC) for predicting up-regulated DEGs was 0.5013 (permutation-based *p* = 0.17), and the prAUC for predicting down-regulated DEGs was 0.499 (permutation-based *p* = 0.22). Therefore, RF models based on known TFBS involved in temperature-related reactions were not able to predict DEGs with high accuracy within this dataset. This, taken together with the various discovered motifs within the upstream and downstream regions of the genes, indicates that a method that incorporates more genetic information within the code might improve the prediction of regulatory status better within specific conditions.

### 3.3. Predicting Gene Expression Regulation Based on Regulatory Regions Using Naive Methods Is Possible

**Figure 2:**
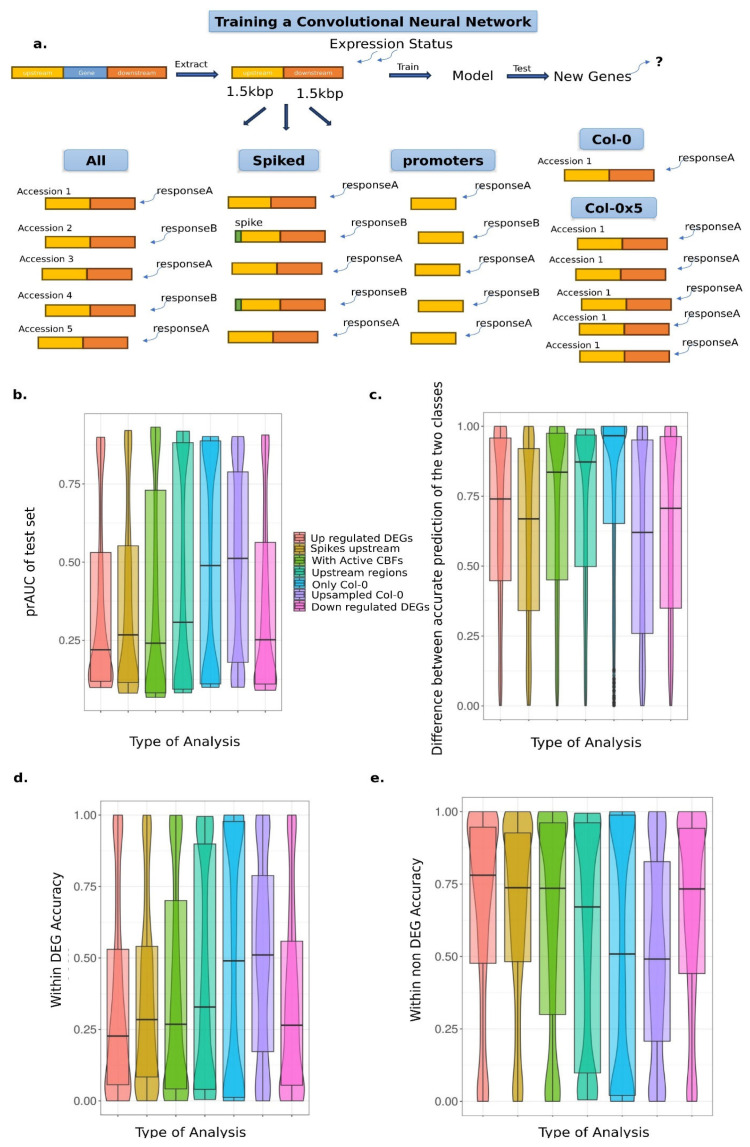
Training convolutional neural networks to predict up-regulated or down-regulated DEGs between specific environments. (**a**) Description of the different types of input data used to train CNNs to predict up- or down-regulated DEGs vs. non-DEGs. The analysis “up-regulated DEGs” included the five *A. thaliana* accessions and both upstream and downstream regions for up-regulated DEGs. The analysis of “spiked upstream” included training on up-regulated DEGs with the first 5 bp being replaced consistently with the same sequence. “With active CBFs” refers to the training of models using the two accessions with all three *CBF* genes active. The analysis of “upstream regions” included only the upstream regions of the up-regulated DEGs as input. “Only *Col-0*” was used to train models, as well as when it was five times oversampled (“*Col-0* × 5”). Finally, the results under the category “down-regulated DEGs” show the outcome of training. (**b**) prAUC values of the test set across different analyses. (**c**) The difference in accurately predicting the up-/down-regulated DEGs to the non-DEGs across different analyses. (**d**) The within-class prediction accuracy of down- and up-regulated DEGs in the test set across all analyses. The within-class prediction accuracy was estimated as the proportion of correctly predicted genes of this class over the total number of genes in this class in the test set. (**e**) The within-non-DEGs prediction accuracy of the test set across all analyses.

We next investigated whether a currently commonly used machine learning method could learn *cis*-regulatory motifs simply from sequence and DE data alone. If de novo motifs can be identified in DE genes, even though known TFBS were not good predictors of expression in the RF model, we might obtain better predictions by learning important motifs de novo. To this end, we trained convolutional neural networks (CNNs) that incorporate the upstream and downstream genetic sequence as an input layer via one-hot encoding (Figure 2a; see Section 2). We trained 1344 models with different combinations of the hidden layer’s parameters to find the model architecture that best predicted the genes DE (Table S2). We trained the models using the information from all five accessions that had available pseudogenomes to predict up- or down-regulated DEGs separately.

The tested CNN model architectures have had a wide variety of accuracy in predicting the DEG of each gene (Table S5). We extracted the prAUC of each model during training. Moreover, we estimated the prAUC of the test set for each trained CNN model. The median and mean prAUC values during training for predicting up-regulated DEGs vs. non-DEGs were 0.5013 and 0.5017, respectively, indicating that many tuning parameter settings did not lead to substantial learning. We also estimated the median within-class accuracy in the test set as 0.776 and 0.227 for non-DEGs and up-regulated DEGs, respectively. Note that the test set is not oversampled, but it is the original ratio, which is mostly non-DEGs. Overall, the median prAUC in the test set of the models was 0.229, with a mean of 0.354 (Figure S2). We identified 15.6% of the models with prAUC in the test set values above 0.80.

To gain insight into whether tuning parameters had any consistent effects on model learning, we used a generalized linear mixed model on the prAUC of the test set using the first layer’s filters as a random effect and the number of convolutional and dense layers, with pool stride and dropout rate as fixed effects. Both the number of convolutional and dense layers had a significant negative impact on the prAUC of the test set, with *p* = 0.000754 and *p* = 3.9708 × 10^−16^, respectively, suggesting more complex models were more poorly trained. Additionally, the pool stride (*p* = 2.918 × 10^−10^), which indicates the number of base pairs that are considered together in each filter, and the dropout rate, or how many model nodes are used each time to predict, had a significant negative impact on the prAUC of the test set. However, none of the interactions between the fixed effects were significant, suggesting little importance of specific combinations of the tuning parameters on learning.

The trained models for predicting down-regulated DEGs or non-DEGs (Figure S3) were very similar to the results observed for the up-regulated DEGs vs. non-DEGs. The median prAUC of the models during training was 0.5009, and the mean prAUC was 0.5012. The median prAUC of the test set for the down-regulated DEGs vs. non-DEGs was 0.253, and the mean was 0.363 (Table S6). The same hyperparameters as for predicting up-regulated DEGs were identified to have a significant negative effect with *p*-values of 1.511 × 10^−7^, 0.0168, 4.068 × 10^−8^, and 0.0485 for number of convolutional layers, number of dense layers, pool stride, and dropout, respectively.

We identified the best models that could predict the up-regulated DEGs. For that purpose, we selected a model that had prAUC in the test set above 0.7. Moreover, in order to understand the models’ potential to predict each class separately, we estimated for each class a prediction accuracy as the number of correct predictions of the class divided by the total number of the genes in the class tested. Hence, after this prAUC cutoff, we picked a model whose prediction accuracy difference was the lowest. The best model for predicting up-regulated DEGs had a prAUC in the test set of 0.701, and prediction accuracy in the test set was 0.368 for up-regulated and 0.693 for non-DEGs (Table 2). The class prediction accuracy difference was 0.325. The best model for predicting up-regulated DEGs had two convolutional layers, two dense layers, a dropout rate of 0.25, and a pool stride of 4. Using the same criteria, we were able to identify the best model in predicting the down-regulated DEGs. The prAUC of the test set for this model is 0.7128, and the difference in the class prediction accuracy is 0.319, with per-class accuracy being 0.365 for down-regulated DEGs and 0.665 for non-DEGs. The model had three convolutional and two dense layers, a pool stride of 8, and a dropout rate of 0.25.

We assessed whether these models performed better than randomness by permuting the input labels and re-training the selected model 100 times (Figure S4) so as to understand to what degree the input labels are used for tuning by the CNN architecture. For each permutation we estimated the prAUC of the test set and the prediction accuracy between the two predicted classes using the un-permuted test set. Then, we compared the values of the original trained model against the distribution of the metrics estimated with the permuted labels. The significantly higher prAUC for the up-regulated DEGs (*p* < 2.2 × 10^−16^) and down-regulated DEGs (*p* < 2.2 × 10^−16^) (when compared to the permuted dataset) indicates that our picked models perform better than randomly picking genes.

### 3.4. The Number of Discovered Motifs Can Impact the Correct Prediction of Up-Regulated DEGs

We investigated the profile of the genes that are accurately predicted within the up-regulated DEGs by the selected model with the highest achieved performance. Out of the 4564 up-DEGs across all five genotypes in the test set, 592 were accurately predicted. The correctly predicted genes had a significantly higher (Wilcoxon rank sum test *p* = 0.018) average number of discovered motifs in their regulatory regions (median of 12 motifs) by the STREME analysis than the genes that were incorrectly predicted (mean of 10; Figure S5a). Interestingly, when we repeated this analysis for the down-regulated DEGs, we did not detect a different accumulation of TFBS within the correctly predicted genes. Both the correctly and wrongly predicted genes had a median of 11 motifs per gene, and the accumulation of the TFBS upstream and downstream of the regions was not significantly different (Wilcoxon sum rank test *p* = 0.9446; Figure S5b). Moreover, the gene expression level of the genes that were correctly predicted vs. not correctly predicted was not significantly different (based on the Kolmogorov–Smirnov test *p* > 0.05) for both down-regulated DEGs and up-regulated DEGs.

Up until this point, the discovered motif diversity, the CNN training results, and even the best model’s behavior were very similar for up-regulated DEGs versus down-regulated DEGs. For the rest of the analyses, we focused on training CNN models for predicting up-regulated DEGs versus non-DEGs, as we do not expect that altering the input of the CNNs’ training will alter the observed pattern between the two regulatory groups.

### 3.5. CNNs Imperfectly Identify a Simple Artificial Signal Within the Regulatory Regions

Because many tuning parameter values resulted in models that learned little (training prAUC near 0.5), we sought to assess potential sources of constraints on these models. We investigated whether the tested model architectures can learn a simple motif within the sequences that perfectly predicts DEGs, a motif we refer to as a “spike”. If these spiked models are perfectly able to predict DEGs in this setting, it suggests that the limitations we observed with real data arise from a lack of such good, simple predictors in the sequence. If models have limited learning in this setting, it suggests that the model architecture is limited in its ability to find signals in the data we are using, perhaps due to the complexity and abundance of sequence data (2000 bp per gene).

To test these hypotheses, we included a spike sequence of 5 bp in the place of the first 5 bp of all up-regulated DEGs (Figure 2a). The spike’s sequence (AAGGG) does not overlap with the sequence of any of the discovered enriched sequences with the up-DEGs. The expectation was that the accuracy of the models would increase as they have a perfect sequence to use for predicting the up-DEG group. During training the prAUC values had a median of 0.5018 and a mean of 0.5046. The median prAUC in the test set of the spiked models was 0.36, with a mean of 0.266, which was on average higher than when trained without the spikes (Table S7). The distributions of the prAUC values in the test set between the spiked and non-spiked models were significantly different (Kolmogorov–Smirnoff test, *p* < 2.2 × 10^−16^; Figure 2b). Generally, the spiked models had fewer overfitted models than the non-spiked models for both up-regulated and non-DEG classes (Figure 2c and Figure S6). In total, 188 spiked models with prAUC values of the test set above 0.8 were detected and 561 models with prAUC values of the test set below 0.2, and thus, in total, 55.7% of the models tested are overfitted to one of the two classes. In contrast, among the non-spiked models, 61.5% of the models are overfitted.

When we used the same criteria to pick the best-performing models for the spiked data (permutation *p* < 2.2 × 10^−16^) as for the non-spiked data, we identified a model that predicts the two classes better than the best non-spiked model. prAUC of the test set (0.704) is only marginally higher by 0.03. In contrast, the difference in the prediction accuracy of the two classes in the test set is 0.185, which is 1.75 times lower than the best model predicting non-spiked up-regulated DEGs. This improvement is due to the higher accurate prediction of the up-regulated DEGs (0.48) in the spiked best model than the non-spiked best model (0.36).

**Figure 3:**
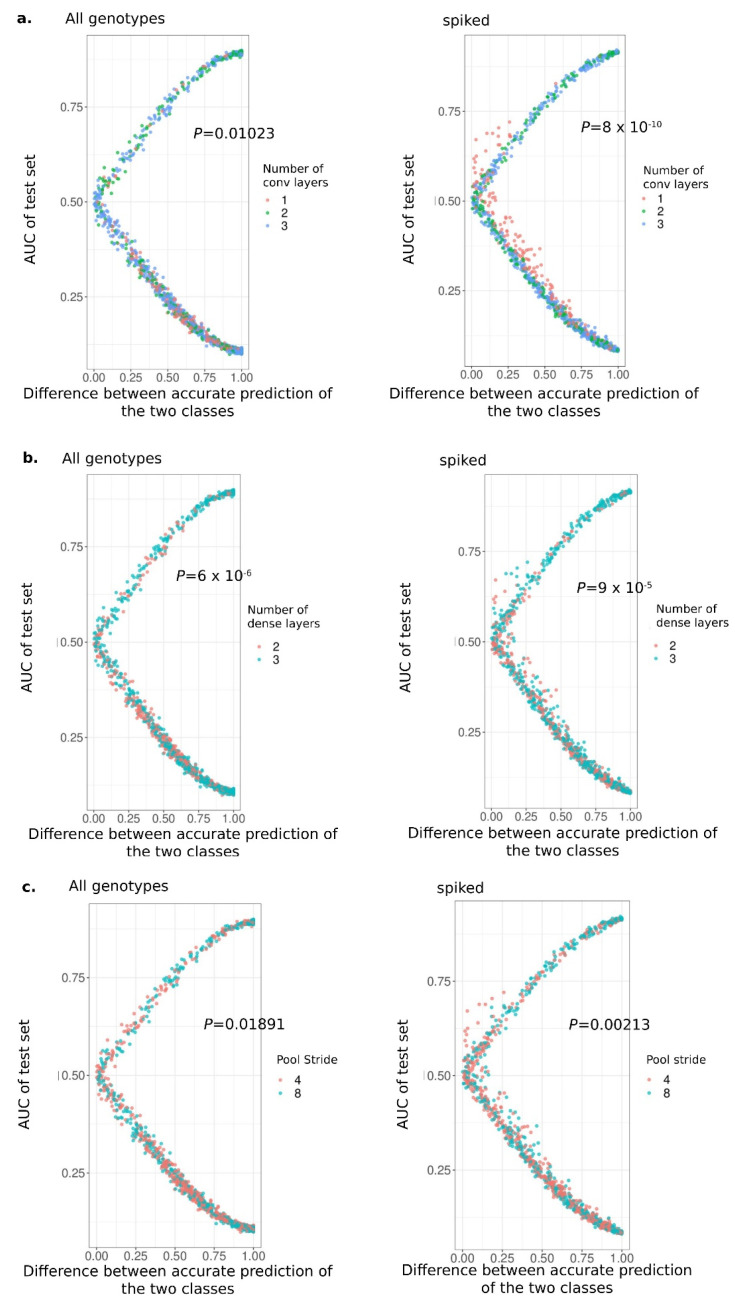
Simple spiked models can accurately predict an introduced spike of 5 bp at the beginning of the up-regulated DEGs promoter region. Comparison of the prAUC in the test set and the difference between the accurate prediction of the two classes for non-spiked (**left**) and spiked (**right**) input data. Comparisons are performed for (**a**) number of convolutional layers, (**b**) number of dense layers, and (**c**) pool stride. The *p*-values stand for the significance level of the KS test when comparing the two distributions shown within each plot.

There is no significant correlation between the prAUC values in the test set of the spiked and non-spiked models under the same values of tuning parameters (*p* = 0.96, ρ = 0.0012), indicating that the inclusion of the spikes did not improve models with good tuning parameters for the real data but instead were best captured with distinct tuning parameters (Figure 3). When we further investigated the different hyperparameters’ impact on the prediction accuracy of each class, we noticed that the spiked models with one convolutional layer had higher accuracy and prAUC in the test set and simultaneously smaller difference between the accurate predictions of both classes than the models with two and three convolutional layers. (Figure 3a). The fact that the models with fewer convolutional layers performed better than those with more of them was also true for the spiked models. A generalized mixed model including the number of convolutional layers, dense layers, pool stride, and dense units as fixed effects indicated that the number of convolutional layers had a significant effect on the difference of the prediction accuracy of the two classes (*p* = 8.091 × 10^−10^). Fewer layers were predicted more accurately, as the slope parameter estimates were negative. This observed pattern is not as extreme as for the non-spiked models (*p* = 0.01023; Figure 3a). Other parameters that were found to have a significant negative impact on the spiked models’ difference in the prediction accuracy of the two classes were the number of dense layers (*p* = 9.036 × 10^−5^), the pool stride (*p* = 0.002137), the first dense layer’s units (*p* = 0.048), as well as the interaction of the number of convolutional and dense layers (*p* = 0.00983). All these factors, except for the interaction of the number of dense and convolutional layers, had a significant effect on the difference between the accuracy of the two classes for the non-spiked models. Specifically, the number of dense layers, the pool stride, and the number of convolutional layers had *p*-values of 6.191 × 10^−6^ and 0.0102, respectively (Figure 3b,c). The estimates for all parameters were negative in both spiked and non-spiked data, which means that generally models with fewer layers were able to predict with higher per-class accuracy in the test set. This result suggests that although different specific sets of tuning parameters are good for spiked data versus the non-spiked, the general effect of tuning parameters, and specifically model complexity, was consistent. Therefore, a consistent signal within the up-regulated DEGs can improve the model performance, indicating that the regulatory complexity among genotypes is quite high, that we lack information in the sequence data for prediction, or that the DEG response is highly stochastic.

### 3.6. Predicting Only Among Genotypes Sharing Alleles for Major Trans-eQTL Does Not Improve the Prediction of the Up-Regulated DEGs

Since the results with the sequence spike indicate some data complexity limited our CNN predictions, we investigated whether we could simplify the prediction task by homogenizing known large-effect *trans* mutations (CBFs). These transcription factors show variation in functionality among genotypes, and in the natural genotypes tested, at least some copies are non-functional or differentially expressed between conditions. We approached this analysis in two ways. First, we trained models predicting up-regulated DEGs vs. non-DEGs in *Rsch*, *Col-0*, and *Ler*, which do not have intronic SNP differences amongst them in all three major well-studied cold-response *CBF* transcription factors, including *CBF2*. With this analysis, we eliminated genetic variation within the *CBFs* in our dataset, which could have altered their function. Then, we trained models to predict up-regulated vs. non-DEGs on *Can* and *Cvi*, which have differential expression of all three *CBFs* between control and cold treatments during this experiment. This way we tested whether their up-regulated status can have an impact on the training.

The median and mean prAUC during training on the set with no SNP differences were 0.5001 and 0.5, respectively, while the prAUC of the test set had a mean value of 0.47 and a median of 0.095 (Figure S7; Table S8). This is also reflected in the percentage of model architectures that have been overfitted for predicting the up-regulated DEGs: 52.5% of the models in contrast to the 45.5% overfitted in predicting the non-DEGs, with only 1.7% of the models not showing any sign of overfitting. The best model trained on only the three accessions with similar copies of CBF functional had prAUC in the test set of 0.705 and a difference in predicting accuracy of the two DEG groups of 0.47. Similarly, the median prAUC during training on the set with all three differentially expressed CBFs was 0.5014, while the prAUC of the test set had a median value of 0.268 (Figure S7; Table S8). The difference in the accurate prediction of the up-regulated DEGs and non-DEGs within the test set has a median of 0.835 and a mean of 0.701, which is on average higher than the difference in prediction accuracy of the models applied to all genotypes. The best model had a prAUC in the test set of 0.7 and a difference in predicting accuracy of the two DEG groups of 0.31. Overall, those models did not perform better than the original models, including all the *A. thaliana* accessions. This suggests that the lack of a major *trans*-acting expression variant did not hinder predictability in our larger set of diverse genotypes.

### 3.7. Including Both Upstream and Downstream Regions Improves the Prediction of Up-Regulated DEG Class

As often the *cis* elements are mostly identified in the upstream regions [63], we tested whether including the downstream coding region limited the prediction accuracy, perhaps by incorporating noise. Noise might be introduced by incorporating long sequences that usually have few TFBS, such as the downstream regions. Therefore, we re-trained all the models by including only the 2k bp upstream regions for all five accessions, compared to the 1 kb up- and 1 kb downstream of the coding region in the approach presented above (Figure 2a). The median prAUC of the training set was 0.5, and the median prAUC (0.307) in the test set of the models trained only using the 2 kb upstream sequences was slightly higher than including both the 1 kb upstream and 1 kb downstream regions, by 0.048 (Figure S8; Table S9). The distribution of the prAUC results in the test set of the two analyses was again significantly different (KS test *p* < 2.2 × 10^−16^). The within-class prediction accuracy of up-regulated DEGs (0.671) in the test set was higher than for non-DEGs (0.328). Moreover, 29.8% of the models had prAUC values in the test set above 0.8. The same pattern was observed for models that had low prAUC in the test set. Approximately 41% of the models had prAUC in the test set below 0.2, indicating models were overfitting by predicting all genes as up-regulated DEGs, for a total of 71.1% of the models overfitting to either class. In comparison, including both upstream and downstream regions leads to overfitting of 61.5% of the models. (Figure 2c,d). Therefore, excluding the downstream regions from the analysis leads to more cases when the models become fixed in recognizing only one class.

The best model in predicting up-regulated DEGs and non-DEGs trained only on putative promoter regions had three convolutional layers, three dense layers, and a dropout of 0.1 (Table 2). The prAUC in the test set was 0.7004, and the difference in accurately predicting up-regulated DEGs and no DEGs is 0.332, which was similar performance to the best model learning using both upstream and downstream regions (Table 2). Its performance was better than models with the same architecture trained on random input (*p* < 2.2 × 10^−16^). Therefore, including only the 2 kb upstream regulatory regions of the genes during training does not significantly alter the predictive ability compared to including 1 kb upstream and 1 kb downstream.

### 3.8. Genetic Diversity Does Not Improve Accuracy of Differentially Expressed Genes

Genetic diversity is an important dimension of natural populations. Incorporating multiple genotypes in the STREME analysis has had a positive impact on discovering more enriched motifs. If we consider the introduced spikes in the upstream region of up-regulated DEGs as large-effect mutations due to their 100% frequency within the group, we also showed that the majority of the information that the models learn is based on small-effect mutations. Small-effect mutations can be better identified when incorporating more genetic diversity into any kind of analysis, as the less frequent variants are easier to identify. We finally compared how effective CNNs were when including multiple genotypes in the training set by training all the different model architectures, including only the most commonly studied *A. thaliana* accession, *Col-0* (Figure 2a). We repeated this twice, once with the original number of genes in *Col-0* and once with up-sampling the *Col-0* genes in order to have the same size of training set as for the complete set of the genotypes. In both cases, we oversampled the up-regulated DEGs to a 1:1 ratio with the no DEGs. The training of the non-oversampled and oversampled sets yielded median prAUC values of 0.5004 and 0.501, respectively.

When compared to the models that have been trained on the *Col-0* dataset only, we see that the median AUC of the test set is higher, as it is at 0.488, for the multiple genotypes model (Figure 2b and Figure S9; Table S10). The accuracy was significantly impacted by the number of replicates, as the models trained on the up-sampled *Col-0* set had a median prAUC of the test of 0.508 (KS test *p* = 8.8 × 10^−16^; Table S11; Figure S10). During training, both datasets had a high proportion of overfitted models. Within the up-sampled dataset, 50.3% of the prAUC values in the test set were either above 0.8 or below 0.2. Training on the up-sampled dataset of *Col-0* had fewer overfitted models than training on the non-oversampled dataset of *Col-0*, in which 77.4% of the models were overfitting in either direction. The better performance of the oversampled dataset of *Col-0* is due to the more appropriate size of the dataset; the original *Col-0* dataset has only 34,002 data points when the up-regulated DEGs are oversampled to a 1:1 ratio [64,65]. Based on this observation, below we compare only the models trained on the oversampled dataset of *Col-0* to the models trained on all five accessions.

The models trained on the *Col-0* dataset have fewer overfitted models (50.3%) than the models trained on all five accessions (61.5%). The best model in predicting both up-regulated and non-DEGs on the *Col-0* dataset had similar architecture and performance statistics as the best model trained on all five accessions. The prAUC value in the test set trained on the oversampled *Col-0* was 0.7034, and the difference in the accurate prediction between the two classes was 0.3122. It performed better than when trained on a permuted dataset (*p* < 2.2 × 10^−16^). Both the prAUC in the test set (0.7008) and the difference in accurate prediction of both classes (0.3252) indicate that training on all five accessions did not perform as well as training on the *Col-0* dataset only. Therefore, including more accessions may either marginally increase the noise and not substantially improve the accuracy of the models, or the additional genotypes’ sequences were not variable enough to capture more contrasts between alleles rather than genes.

Finally, because the models of cold response had only modest success, we tried to test if the CNN models could accurately predict DEGs in a simpler transcriptomic change (1282 DEGs), based on a single transcription factor knockout known to control anthocyanin biosynthesis. However, even these CNN predictions had modest success (Supplementary Materials), highlighting the challenge in predicting transcriptomic dynamics from proximate DNA sequences.

## 4. Discussion

We used the genomic sequence of upstream and downstream regions of *A. thaliana* genes to identify motifs related to the differential expression in cold and control conditions and also to predict the gene expression response to an abiotic stressor. Many *cis*-regulatory elements have been documented [13,38,66], and they are often involved in gene evolution and adaptation to novel environments [4,67]. Here, within both the up-regulated and down-regulated DEGs, we found motifs of variable length that are enriched within them in comparison to the non-DEGs. The discovered motifs in regulatory regions significantly overlap with known transcription factor binding sites that are involved in the regulation of gene expression under cold stress [42,62,68]. Knowledge of specific transcription factor binding sites can aid the prediction of gene expression under different types of abiotic and biotic stress [27,33,69,70]. The five accessions used in this study are regulated by differential function of *CBF* and *ZAT12* regulons, leading to their variable acclimation capacity to cold [42]. The three *CBF* genes control the acclimation to cold conditions and reaction to freezing stress [58,71]. It has been shown that genetic diversity within the gene and its network is connected to adaptation to different temperatures and latitudes [9,45,72]. We identified a frequent overlap with known TFBS, which are known to be involved in controlling responses to cold or freezing stress, such as the RVEs [73], members of the DOF family of transcription factors [58,62,74,75], and FLC [76]. We also identified frequent binding sites for members of the DREAM complex, which are involved in repressing growth in response to DNA damage [77], as well as SGR5, which is differentially spliced under heat stress [78]. This indicates that it may be possible to predict gene expression dynamics using not only molecular biological features but also genetic diversity in sequence and expression, as the incorporation of more accessions than *Col-0* yielded more discovered motif sequences.

However, as often the available information to the researcher is only DNA and mRNA sequence (and not experimentally verified transcription factor binding sites), we investigated the possibility of using the genetic sequence to predict gene expression. The need for this was also evident by trying to predict up- or down-regulated DEGs using random forest analysis. The random forest used the known presence or absence of specific TFBS upstream and downstream of the genes to predict DEGs. However, neither for up-regulated DEGs nor for down-regulated DEGs was the prediction accuracy better than chance. In *A. thaliana*, using known transcription factor binding sites has not yielded very good prediction of differential gene expression [27], while the most enriched *cis*-regulatory elements did not also predict the most differentially expressed genes in maize [32]. Training CNNs with the 2k bp upstream and downstream regions of the genes yielded better prediction accuracy. The model that had both high accuracy and had the least difference in predicting accuracy per class had prAUC in the test set of 0.7. This value is slightly lower than what has been reported in similar studies in other species, which identified models with prAUC between 75% and 85% [55,79]. In our case, higher prAUC values showed signs of overfitting; non-DEGs, which comprise more than 80% of the dataset, were then almost exclusively correctly predicted, inflating the accuracy and prAUC. Surprisingly, the inclusion of both upstream and downstream putative regulatory regions did not improve the prediction accuracy of DEGs, as in maize [33]. This could be attributed to genomic differences between the species; for instance, downstream regulatory regions have differences in nucleotide composition between maize and *A. thaliana* [80]. Additionally, maize has a different genomic composition than *A. thaliana*, such as higher transcription element density, with 85% in contrast to 21% in *A. thaliana.* Authors have suggested the expanded genome of maize could enhance the landscape and availability of adaptive *cis*-regulatory mutations [81]. Factors such as this can explain the difficulty of successfully transferring machine learning protocols generated from one species to another.

Phenotypic responses to the environment (plasticity) can be adaptive, which may ultimately be fixed by populations in specific environments [82]. Phenotypic plasticity can also be genetically variable (i.e., GxE), with gene expression being an important example. Our hypothesis was that incorporating more genotypes in this study would improve the prediction accuracy, as more genetic variation in *cis* sequences and expression would be incorporated during training. Incorporating genetic variability between species has been successfully used to train models with simple architecture to predict gene network relationships [83]. Within maize, when more than one genotype was used for predicting gene expression, then the results were more accurate [33]. We did not observe the same pattern here; in fact, an oversampled dataset of *Col-0* upstream and downstream regions to the same size as the dataset including the multiple genotypes yielded a model with higher prAUC and more comparable prediction of the two DEG classes. We do not believe that this is simply an issue of power due to the dataset being on the smaller side of what is required for machine learning. A potential contributing factor could be that the *cis*-regulatory genetic variation might not be of large enough frequency between genotypes, but this variation is stronger between genes. Additionally, the highest prAUC in the test set was achieved when the accessions with all three differentially expressed *CBF* genes were only included during training, suggesting *trans*-acting variants at high frequency could limit our predictions across multiple genotypes. This pattern was confirmed when a high-frequency motif was incorporated in the upstream regions of the up-regulated DEGs; the spiked dataset could be used to train models with higher accuracy. Within *A. thaliana*, the *CBF/DREB* genes of the cold signaling pathway have been linked with cold adaptation within Europe [44,84] as the major eQTLs controlling acclimation. However, there are possibly more *trans*-acting effects, which might confound and impact the predictions and were not taken into consideration here. Taken together with the fact that more than half of the enriched discovered motifs within the up- and down-regulated DEGs are present in less than 50% of the genes across all genotypes, and thus, not very frequent, it indicates that the large number of *cis*-regulatory variants across the genome in low frequency can decrease the accuracy in prediction of gene expression differences, in contrast to the presence of a potential large effect eQTL controlling the response to cold. The putative promoter region likely captures the many *cis*-regulatory elements, which have been related to large impacts on gene expression divergence and polygenic selection but may often each individually be mutations of small effect [66,85]. Incorporating a spike within the upstream region, which represents a single factor of large effect, supports this hypothesis, as the spike improved the prediction accuracy of the models overall. Our findings are consistent with machine learning predictions of protein folding, where models that accurately predict variation among genes perform worse on predicting effects of individual amino acid substitutions [86], a task that was better suited to a distinct prediction approach [87]. During training, the models were able to detect the spike and increase the accuracy of the up-regulated DEGs. Therefore, even though including multiple accessions could slightly increase the accuracy of predicting GxE, care has to be taken as to which accessions are selected for the training.

One explanation for the remaining variation in expression unpredicted by CNNs in this study is that these models overlook much of the biological complexity driving gene expression response to environments. Genes are expressed as a part of complicated regulatory networks that *cis*-regulatory elements only partially capture. We have currently ignored epigenetic states and the sequence of the coding region. In a sorghum species, it has been more successful to predict DEGs as a response to cold when models incorporated information about the coding region sequence and methylation information [79]. Alongside specific transcription factor binding sites, their positions on the genome and the chromatin state have been useful to predict the transcription factor’s target genes, with information that is also transferable with high accuracy between datasets [88]. Prediction accuracy of gene expression differences and the impact of *cis* variants were improved when large up- and downstream regions, as well as unmethylated regions, were included in the analysis [32,33]. Trained bimodal CNNs based on chromatin state and DNA sequence for different cell types can differentially predict the transcription factor binding specificity, which performs better than neural networks based only on DNA sequence information [89]. During training, therefore, using only *cis*-regulatory regions to predict gene expression response to environment within *A. thaliana* may be of limited accuracy without incorporating additional data types, such as co-expression networks, distal enhancers, and epigenetic states. However, when that biological information is missing and it is challenging to acquire, as would be the case in many non-model systems, the accuracy of imperfect CNN models might still be of use to the evolutionary researcher.

Interpretation of what might have been having an impact on the training could be difficult to understand. Even models with the best performance do not necessarily have the best interpretable structure of layers [90]. Looking into the statistics of all the different models trained during each grid search can give insights into this. First, the distribution of the prAUC during testing can give clear indications of how easy it is to overfit the model and, therefore, how complex this training task can be. Despite these limitations, using machine learning methods can enable researchers to draw predictions and conclusions about reactions to specific environments within species, albeit perhaps not CNN models. We believe that the plant evolutionary and ecological communities would benefit from further development and use of other types of models, such as language models, which can use less biological information to predict gene expression responses. Those models would be especially useful for systems with less information about their (molecular) biology than the model species.

## Figures and Tables

**Table 1:** The top known transcription factor binding sites (TFBSs) that significantly overlap with the enriched discovered motifs along the upstream and downstream regions of up- and down-regulated regions of up- and down-DEGs.

TFBS	Number of Discovered Motifs	Gene	Family
MA1159.1	4	*SGR5*	C2H2 zinc finger factors (class)
MA1268.1	3	*CDF5*	DOF
MA0556.1	3	*DOF1.7*	DOF
MA0558.1	3	*FLC*	MIKC
MA0559.1	3	*(PISTILLATA) PI*	MIKC
MA0940.1	3	*AP1*	MADS box factors (AP1)
MA1085.1	3	*WRKY40*	WRKY (class)
MA1281.1	3	*DOF5.1*	DOF
MA1367.1	3	*AT1G76870*	Trihelix
MA1380.1	3	*TCX6*	CPP
MA1182.1	5	*RVE8*	Myb-related
MA1184.1	5	*RVE1*	Myb-related
MA1380.1	5	*TCX6*	CPP
MA1277.1	4	*DOF1.7*	DOF
MA1281.1	4	*DOF5.1*	DOF
MA1268.1	4	*CDF5*	DOF
MA1278.1	4	*DOF3.4*	DOF
MA1279.1	4	*DOF1.5*	DOF
MA1190.1	4	*RVE5*	Myb-related
MA0933.1	4	*AHL20*	HMGA factors
MA1267.1	4	*DOF5.8*	DOF

**Table 2:** The best model for each performed training. For each type of analysis run (Figure 2), we selected the best-performing model based on the prAUC value of the test set and the difference in accurately predicting the DEGs vs. non-DEGs. For each model, the model parameters; the training, validation, and testing prAUC; and loss and accuracy of predicting both DEG classes are given below.

	Up-regulated DEGs	Down-regulated DEGs	Spiked Up-regulated DEGs	Upstream Regions of up-DEGs	Up-regulated DEGs of Only *Col-0*	Up-regulated DEGs of Up-sampled *Col-0*	Genotypes with All *CBFs* as DEGs
1st conv. filters	128	64	128	64	128	128	128
2nd conv. filters	128	64	None	128	None	128	128
3rd conv. filters	None	128	None	128	None	None	64
Conv. width	4	4	4	4	4	8	8
Pool width	4	4	8	4	4	4	4
Pool stride	4	8	4	4	8	4	8
Dropout	0.25	0.25	0.25	0.1	0.1	0.1	0.25
1st dense layer units	64	128	128	128	128	128	64
2nd dense units	128	None	128	None	128	128	None
Number of conv. layers	2	3	1	3	1	2	3
Number of dense layers	3	2	3	2	3	3	2
Learning rate	0.0001	0.0001	0.0001	0.0001	0.0001	0.0001	0.0001
Loss	0.692	0.691	0.691	0.691	0.691	0.692	0.691
Accuracy	0.621	0.682	0.673	0.666	0.692	0.636	0.672
Accuracy of DEGs	0.368	0.365	0.480	0.339	0.347	0.368	0.341
Accuracy of non-DEGs	0.693	0.684	0.665	0.671	0.697	0.681	0.681
prAUC test set	0.701	0.712	0.704	0.701	0.7207444	0.703	0.711
prAUC training	0.499	0.502	0.522	0.499	0.4971713	0.505	0.499
prAUC of the validation set	0.501	0.502	0.578	0.509	0.4851478	0.517	0.506
Loss of the validation set	0.693	0.693	0.692	0.693	0.693379	0.693	0.693
Epoch of the best model	37	50	49	29	49	48	49
Difference in accuracy of DEG classes	0.325	0.319	0.185	0.332	0.3495906	0.312	0.340

## Data Availability

All scripts are available at the GitHub repositories: https://github.com/mtakou/cnns_DEGpromoters (accessed on 1 November 2021) and https://github.com/em-bellis/CNN_GxE.git (accessed on 1 September 2024).

## Supplementary Materials

The following supporting information can be downloaded at: https://www.mdpi.com/article/10.3390/genes16091108/s1, Figure S1: Known transcription factor binding sites of Col-0 poorly predict the expression response to cold, with PRAUC = 0.503; Figure S2: Summary statistics of all models trained during the grid search for predicting up-regulated DEGs vs non DEGs. Figure S3: Summary statistics of all models trained during the grid search for predicting downregulated DEGs vs non DEGs. Figure S4: Distribution of the prAUC in the test set for the permuted datasets of a) up-regulated DEGs (up) and b) down-regulated prAUC; Figure S5: The average number of motifs per gene for the correctly predicted (blue) and not correctly predicted DEGs (red) by the best model predicting a. up-regulated DEGs or b. down regulated DEGs. Figure S6: Summary statistics of all models trained during the grid search for predicting up-regulated DEGs vs no DEGs, while including a spiked region at the start of the up-regulated genes’ putative promoters. Figure S7: Summary statistics of all models trained during the grid search for predicting up-regulated DEGs vs non DEGs, while using the two accessions with all 3 CBF genes differentially expressed between the two treatments (red) and all 3 CBF genes having no SNP differences between the accessions. Figure S8: Summary statistics of all models trained during the grid search for predicting up-regulated DEGs vs non DEGs, while including only up-regulated genes’ putative promoters. Figure S9: Summary statistics of all models trained during the grid search for predicting up-regulated DEGs vs no DEGs, while including only *Col-0*. Figure S10: Summary statistics of all models trained during the grid search for predicting up-regulated DEGs vs no DEGs, while including only an up sampled set of *Col-0*. Figure S11: Summary statistics of all models trained during the grid search for predicting up-regulated DEGs vs no DEGs in an up sampled set of a tt8 knock out dataset. Table S1: Information about the number of down regulated, no differentially regulated, and up regulated genes within the five accessions as described in Hannah et al. 2006 [42]. Table S2: The hyperparameter combinations used during grid search training of the CNN models. Table S3: Discovered motifs upstream and downstream of all the up-regulated genes. Table S4: Discovered motifs upstream and downstream of all the down-regulated genes. Table S5: Results of training 1356 CNN models on all genotypes upstream and downstream regions to predict up DEGs vs no DEGs. Table S6: Results of training 1356 CNN models on all genotypes upstream and downstream regions to predict down DEGs vs no DEGs. Table S7: Results of training 1356 CNN models on all genotypes spiked upstream and downstream regions to predict up DEGs vs no DEGs. Table S8: Results of training 1356 CNN models on Can and Cvi (which have all CBF genes active) upstream and downstream regions to predict up DEGs vs no DEGs. Table S9: Results of training 1356 CNN models on only upstream regions to predict up DEGs vs no DEGs. Table S10: Results of training 1356 CNN models on Col-0 upstream and downstream regions to predict up DEGs vs no DEGs. Table S11: Results of training 1356 CNN models on the up sampled Col-0 upstream and downstream regions to predict up DEGs vs no DEGs.
